# The impact of grain type and reduced crude protein formulation on postprandial glucose and amino acid responses

**DOI:** 10.1016/j.psj.2026.107591

**Published:** 2026-08-07

**Authors:** Mengzhu Wang, Shemil P. Macelline, Mehdi Toghyani, Sonia Y. Liu

**Affiliations:** a School of Life and Environmental Sciences, Faculty of Science, The University of Sydney, Sydney, NSW, 2006, Australia; b Poultry Research Foundation, The University of Sydney, Camden, NSW, 2570, Australia

**Keywords:** Fat pad, Glucose, Insulin, Low protein diet, Starch

## Abstract

Two experiments were conducted to evaluate the post‑prandial glucose, insulin, and amino acid responses in broiler chickens offered diets containing standard and reduced crude protein (CP) levels. Exp. 1 used 120 straight-run Ross 308 broilers to compare standard (SCP) versus reduced crude protein (LCP) wheat‑based diets from 10 to 38 days post‑hatch where growth performance, starch and amino acid digestibility, carcass traits, abdominal fat deposition, and post‑prandial glucose and insulin dynamics were assessed. Exp. 2 used a 2 × 2 factorial design to examine the interactive effects of dietary crude protein level and grain type (wheat or sorghum) on post‑prandial glucose, insulin, plasma amino acids in 144 straight-run Ross 308 broilers from 14 to 35 days post‑hatch. In Exp. 1, the LCP diet reduced weight gain and increased feed conversion during the grower phase and decreased breast yield but did not affect finisher or overall performance. Increased starch intake, starch disappearance rates, starch‑to‑protein disappearance ratios and nitrogen retention were observed in birds offered LCP diet. Amino acid digestibility in the jejunum and ileum was generally enhanced under the LCP diet. In Exp. 2, wheat-based diets led to higher glucose concentrations at 30 min, and there was a treatment effect on postprandial insulin response. Wheat-based diets exhibited higher circulating Val and Ile but not Leu at 120 and 200 min regardless of dietary CP levels and LCP diets increased plasma glutamine concentrations and total area under the curve for glutamine. Overall, no clear relationship between post-prandial amino acid concentrations and growth performance was observed; no clear correlation between glucose, insulin, and fat pad weights was observed, which differed from our hypotheses. Reducing dietary crude protein with NBAA supplementation improved nitrogen utilisation and apparent amino acid digestibility; however, these benefits did not consistently result in improved growth performance.

## Introduction

Understanding the mechanisms behind excessive fat deposition in reduced crude protein (CP) diets remains an ongoing challenge. In broiler chickens offered reduced CP diets, excessive fat accumulation in reduced CP diets is often positively correlated with feed conversion ratio (FCR) (Chrystal et al., 2021). Even in cases where dietary CP is successfully reduced without compromising growth performance, increased abdominal fat pad deposition has been observed. For example, Chrystal et al. (2021) reported that reducing dietary CP from 222 to 165 g/kg in maize-based diets did not affect FCR (1.453 vs. 1.473 g/g; P > 0.05), but abdominal fat pad weight increased significantly from 6.4 to 12.8 g/kg (P < 0.001). Similarly, Wang et al. (2025) found that reducing dietary CP from 205 to 175 g/kg did not influence weight gain (2122 vs. 2096 g/bird) or FCR (1.450 vs. 1.464 g/g) between 14 - 35 days post-hatch, but abdominal fat pad weight increased from 8.01 to 10.89 g/kg (P < 0.001).

The role of surplus energy supply in increased fat deposition in reduced CP diets was investigated and reducing dietary energy did not alter fat pad deposition in reduced CP diets. Macelline et al. (2023) examined the effect of reducing dietary energy from 13.0 to 12.5 MJ/kg in reduced CP diets and found that lowering CP from 210 to 170 g/kg increased abdominal fat pad weight from 5.89 to 8.68 g/kg (P < 0.001), while reducing dietary energy by 0.5 MJ/kg had no significant impact on fat pad weight (7.34 vs. 6.90 g/kg; P = 0.369). Hence, dietary energy density *per se* is less likely to be the primary cause of increased fat deposition in broilers offered reduced CP diets. Both starch intake and elevated starch digestion rate may contribute to excessive fat deposition and a series of studies have attempted to manage carcass composition in broiler chickens by restricting the dietary starch-to-protein ratio; however, the results remain inconclusive (Greenhalgh et al., 2020; 2022). The rationale was elevated quantity and rate of starch digestion may lead to hyperglycemia and hyperinsulinemia, which could increase *de novo* lipogenesis and fat deposition (Norton et al., 2022). The relevance of insulin in low-protein diets was discussed in Selle et al. (2025) where the authors proposed that starch digestion differences in reduced CP diets based on wheat and maize may lead to modifications of the anabolic impact of insulin. Despite such concept being well studied in mammalian species, its relevance in poultry remains unclear.

Studies in mice demonstrate that rapidly digestible starch promotes greater adiposity compared with slowly digestible or resistant starch, which reduces fat accumulation and improves metabolic outcomes (So et al., 2007; Solon-Biet et al., 2014). Insulin has been related to overweight and obesity in humans (Kolb et al., 2018). However, the role of insulin in avian species has been dismissed because birds are inherently hyperglycemic and thought to be insulin resistant (Braun and Sweazea, 2008). It was reported that broiler chickens are sensitive to insulin to about 21 days of age but then become increasingly insulin resistant (Franssens et al., 2014).

Postprandial nutrient responses play a critical role in determining energy partitioning between protein accretion and fat deposition. Following a meal, elevations in plasma glucose and amino acids may stimulate insulin secretion, thereby promoting anabolic pathways, including lipogenesis. Most studies in the literature on insulin and glucose responses in poultry rely on glucose‑challenge models, in which a high dose of glucose is either injected or orally administered. The impact of real, complete practical poultry feeds has rarely been studied. Moreover, Emmans (1987) suggested feed intake is regulated and growth is constrained by the supply of the first‑limiting nutrient, not simply energy. Balancing the amino acid profile in reduced CP diets is critical but remains challenging (Siegert et al., 2025). When the balance of amino acids is inadequate, protein synthesis may not proceed at its potential rate despite sufficient energy intake (Liu and Selle, 2017). Under these conditions, surplus dietary energy, which cannot be utilised for lean tissue accretion, may be directed towards lipid deposition. Certain amino acids, including leucine and arginine are also known to directly influence insulin secretion (Thams and Capito, 1999; Yang et al., 2012). Hence, assessing the effects of reduced CP diets on amino acid digestibility and postprandial absorption may provide further insight into increased fat deposition in broiler chickens.

Therefore, two experiments were conducted using diets reduced by 3 percentage points in CP (N × 6.25) compared with the control. In the present study, reduced CP diets are defined as formulations in which non-bound amino acids are included to meet amino acid requirements while lowering overall dietary crude protein levels. Consequently, the reduction in CP is achieved alongside adjustments to other dietary components, including soybean meal, cereal grains, and oil inclusions, as determined by least-cost feed formulation. Although the term “reduced CP diets” is used to describe treatment effects, it should be recognised that these diets reflect comprehensive changes in ingredient composition rather than a simple reduction in crude protein alone. Experiment 1 aimed to test the hypothesis that when balancing amino acid profile, such a moderate reduction in CP would not compromise growth performance, and that amino acid digestibility may increase with greater inclusion of supplemental non‑bound amino acids. Postprandial glucose and insulin responses were assessed, along with the association between amino acid digestion rates, as indicated by apparent jejunal digestibility, and their relationship with abdominal fat deposition. The null hypothesis was that reducing dietary CP would not influence amino acid digestibility or postprandial glucose and insulin responses.

Following the observations from Experiment 1, where reducing CP did not alter fat pad deposition in broiler chickens fed wheat‑based diets, a second study was conducted in addition to the growth performance component (Wang et al., 2025). Consistently, reducing CP in wheat-based diets did not increase fat deposition, whereas an increase was observed in sorghum-based diets. To better understand this interaction, Experiment 2 evaluated postprandial glucose, insulin, fatty acid, amino acid, and ammonia responses in broiler chickens, using fewer sampling time points. The null hypothesis was that grain type (wheat or sorghum) would not influence postprandial metabolite responses under reduced CP conditions. In both studies, correlations between glucose and insulin responses, and their associations with fat deposition and growth performance, were also discussed.

## Methodology

### Materials and methods

These two studies complied completely with specific guidelines (Project No. 2023/2316) that were authorized by the Research Integrity and Ethics Administration of The University of Sydney.

#### Experiment 1 design and birds management

A total of 120 Ross 308 straight-run broiler chickens were used in this study. The experiment consisted of two treatments: standard crude protein diets (SCP) and reduced or low crude protein diets (LCP). Table 1 reported the experimental diets composition and nutrient specifications. The chicks were procured from a commercial hatchery and initially offered a common starter with 12.5 MJ/kg AME and 12.8 g/kg digestible lysine. At 10 days post-hatch, birds were individually identified, weighed, and distributed amongst 20 bioassay cages (750 mm in width, 750 mm in length and 510 mm in height) by body weights so that cage body weight means and variations within cages were statistically similar, the mean body weight was 307 ± 6.8 g/bird at 10 days post-hatch. Each of the two experimental diets were offered to broiler chickens from 10 to 24 days post-hatch as grower, and 25 to 38 days post-hatch as finisher. Each treatments included 10 replicate cages with six birds per cage. Birds had unlimited access to feed and water in an environmentally controlled facility with a 23-hours-on-1-hour-off lighting regime for the first three days post-hatch and 18-hours-on-6-hour-off for the remainder of the study. An initial room temperature of 32 ± 1°C was maintained for the first week and was gradually decreased to 22 ± 1°C by the end of the third week and maintained at this temperature for the remainder of the feeding study.

**Table 1:** Composition and nutrient specifications in grower and finisher diets in Exp 1.

Ingredients %	SCP^1^	LCP	SCP	LCP	Nutrient %	SCP	LCP	SCP	LCP
	Grower		Finisher			Grower		Finisher	
Wheat 10.7 %	64.7	77.3	63.0	76.2	AME (kcal/kg)	3050	3050	3100	3100
SBM 47.0 %	29.9	16.0	28.4	15.2	DM	90.2	90	90.5	90.2
Canola oil	2.36	0.50	4.08	1.95	CP	21.5	18.5	20.5	17.5
Lime fine 38 %	1.01	1.03	1.04	1.06	Lys^6^	1.18	1.18	1.08	1.08
MDP 2	0.60	0.80	0.22	0.40	Met	0.58	0.64	0.54	0.59
Na_2_CO_3_	0.22	0.48	0.20	0.51	Met+Cys	0.92	0.92	0.86	0.86
Lys-HCl	0.25	0.67	0.17	0.57	Thr	0.83	0.83	0.76	0.76
DL-Met	0.30	0.41	0.26	0.37	Ile	0.84	0.8	0.81	0.75
L-Thr	0.15	0.34	0.11	0.28	Leu	1.47	1.3	1.41	1.19
L-Arg	0.00	0.38	0.00	0.30	Trp	0.26	0.19	0.25	0.18
L-Ile	0.00	0.19	0.00	0.15	Arg	1.31	1.3	1.25	1.19
L-Leu	0.00	0.19	0.00	0.11	His	0.5	0.38	0.48	0.37
Gly	0.00	0.27	0.00	0.20	Val	0.91	0.91	0.87	0.84
L-Val	0.00	0.23	0.00	0.19	Phe	0.93	0.7	0.89	0.68
L-Pro	0.00	0.01	0.00	0.03	Gly	0.74	0.84	0.71	0.76
Premix^3^	0.20	0.20	0.20	0.20	Ser	1	0.78	0.96	0.76
K_2_CO_3_	0.00	0.14	0.00	0.04	Pro	1.38	1.25	1.33	1.25
Chloride 60 %	0.05	0.12	0.04	0.10	Ala	0.74	0.54	0.71	0.52
Salt	0.25	0.08	0.25	0.04	Asp	1.65	1.09	1.58	1.06
Sand	0.00	0.70	0.00	0.00	Glu	4.49	3.92	4.33	3.83
Xylanase 4	0.02	0.02	0.02	0.02	Crude fat	3.78	2.03	5.44	3.44
Phytase 5	0.02	0.02	0.02	0.02	Crude fibre	2.64	2.38	2.54	2.33
Celite	–	–	2.00	2.00	Starch	41.1	48.9	40	48.2
Total NBAA	0.64	2.55	0.51	2.08	Calcium	0.8	0.8	0.75	0.75
					Avg. P	0.45	0.45	0.38	0.38
					Total P	0.52	0.5	0.43	0.41
					DEB^7^	219	190	212	180
					*Analysed*				
					CP	21.9	18.9	21.3	17.9
					Digestible CP	18.0	15.9	17.5	15.0
					Starch	37.6	43.4	36.5	44.3

1 SCP = standard crude protein diet, LCP = low crude protein diet.

2 MDP Mono Dicalcium Phosphate.

3 Vitamin concentrate supplied per kilogram of diet: retinol, 12000 IU; cholecalciferol, 5000 IU; tocopheryl acetate, 75 mg, menadione, 3 mg; thiamine, 3 mg; riboflavin, 8 mg; niacin, 55 mg; pantothenate, 13 mg; pyridoxine, 5 mg; folate, 2 mg; cyanocobalamin, 16 μg; biotin, 200 μg; cereal-based carrier, 149 mg; mineral oil, 2.5 mg. Trace mineral concentrate supplied per kilogram of diet: Cu (sulphate), 16 mg; Fe (sulphate), 40 mg; I (iodide), 1.25 mg; Se (selenate), 0.3 mg; Mn (sulphate and oxide), 120 mg; Zn (sulphate and oxide), 100 mg; cereal-based carrier, 128 mg; mineral oil, 3.75 mg.

4 Xylanase (Danisco, Dupont Nutrition & Bioscience) 40,000 U/g.

5 Phytase (Axtra PHY, Dupont Nutrition & Bioscience) 10,000 FTU.

6 All amino acids are expressed on standardized ileal digestible basis.

7 DEB: dietary electrolyte balance = K^+^ + Na^+^- Cl^−^.

#### Experiment 2 design and birds management

The experimental design consisted of a 2 × 2 factorial array of treatments with two levels of dietary CP (205 versus 175 g/kg) and two feed grains (wheat or sorghum), which were offered to a total of 144 Ross 308 straight-run broiler chickens from 14 to 35 days post-hatch. Table 2 showed the experimental diets composition and nutrient specifications. The chicks were procured from a commercial hatchery and offered a same common starter with Experiment 1 from 0 to 13 days post-hatch. At 14 days post-hatch, birds were individually identified (wing-tags), weighed and distributed amongst 24 bioassay cages. The mean body weight of the 24 cages was 548 ± 4.5 g/bird at 14 days post-hatch. Each of four dietary treatments were offered to 6 replicate cages (six birds per cage) to 35 days post-hatch. Feeding, light and temperature regimes followed the Exp. 1. The performance and digestibility results has been published in Wang et al. (2025) and the present study focuses on post-prandial glucose and amino acid responses.

**Table 2:** Composition and nutrient specifications in diets in Exp 2.

Ingredients %	Wheat		Sorghum		Nutrient %	Wheat		Sorghum	
	SCP^1^	LCP	SCP	LCP		SCP	LCP	SCP	LCP
Wheat 12 %	64.7	82.2			Dry matter	91.2	91.2	90.0	89.9
Sorghum 10 %			62.3	74.5	AME MJ/kg	12.9	12.9	12.9	12.9
Soybean Meal 46.0 %	25.9	6.6	28.2	13.1	Crude Protein	20.5	17.5	20.5	17.5
Canola Oil	4.02	1.02	3.94	2.13	Starch	41.0	51.8	39.5	47.1
D,L-Met	0.34	0.41	0.35	0.39	Glu	4.19	3.50	3.21	3.20
Gly	0.00	0.51	0.00	0.49	Lys^6^	1.12	1.12	1.12	1.12
L-Arg	0.11	0.63	0.10	0.53	Met	0.58	0.57	0.62	0.60
L-His	0.00	0.17	0.00	0.15	TSAA	0.89	0.89	0.89	0.89
L-Ile	0.04	0.34	0.00	0.24	Thr	0.75	0.75	0.75	0.75
L-Leu	0.00	0.46	0.00	0.10	Val	0.85	0.85	0.85	0.85
Lys-HCl	0.29	0.73	0.27	0.63	Ile	0.77	0.77	0.77	0.77
L-Phe	0.00	0.31	0.00	0.23	Leu	1.26	1.23	1.49	1.23
L-Thr	0.17	0.42	0.14	0.34	Arg	1.21	1.21	1.21	1.21
L-Trp	0.00	0.04	0.00	0.04	His	0.43	0.43	0.42	0.43
L-Val	0.06	0.36	0.02	0.26	Trp	0.23	0.18	0.21	0.18
L-Cys	0.00	0.08	0.00	0.08	Tyr	0.66	0.52	0.83	0.58
L-Glu	0.00	0.10	0.00	0.88	Gly equivalents^7^	1.49	1.45	1.45	1.45
L-Pro	0.00	0.17	0.00	0.21	Phe	0.85	0.84	0.87	0.84
Limestone	1.17	1.21	1.14	1.10	Calcium	0.80	0.80	0.80	0.80
MDP^2^	0.42	0.62	0.53	0.92	Available P	0.40	0.40	0.40	0.44
Potassium Carbonate	0.00	0.47	0.00	0.39	Total P	0.50	0.48	0.43	0.44
Sodium Bicarbonate	0.28	0.58	0.35	0.61	Crude fibre	2.50	2.15	2.47	2.15
Vitamin-min. premix^3^	0.20	0.20	0.20	0.20	Crude fat	5.47	2.64	5.99	4.40
Choline chloride 75 %	0.06	0.12	0.12	0.18	Sodium	0.19	0.19	0.19	0.19
Xylanase^4^	0.02	0.02	0.02	0.02	Potassium	0.73	0.72	0.75	0.74
Phytase^5^	0.01	0.01	0.01	0.01	Chloride	0.24	0.23	0.24	0.24
Celite	2.00	2.00	2.00	2.00	DEB, (mEq/kg)^8^	202	200	206	205
Salt	0.20	0.00	0.17	0.00	*Analysed*				
Total NBAA	0.95	4.58	0.82	4.45	CP	19.8	16.7	20.5	18.0
					Digestible CP	17.9	15.1	17.7	14.9
					Starch	38.4	48	43	30

1 SCP = standard crude protein diet, LCP = low crude protein diet.

2 MDP Mono Dicalcium Phosphate.

3 Vitamin concentrate supplied per kilogram of diet: retinol, 12000 IU; cholecalciferol, 5000 IU; tocopheryl acetate, 75 mg, menadione, 3 mg; thiamine, 3 mg; riboflavin, 8 mg; niacin, 55 mg; pantothenate, 13 mg; pyridoxine, 5 mg; folate, 2 mg; cyanocobalamin, 16 μg; biotin, 200 μg; cereal-based carrier, 149 mg; mineral oil, 2.5 mg. Trace mineral concentrate supplied per kilogram of diet: Cu (sulphate), 16 mg; Fe (sulphate), 40 mg; I (iodide), 1.25 mg; Se (selenate), 0.3 mg; Mn (sulphate and oxide), 120 mg; Zn (sulphate and oxide), 100 mg; cereal-based carrier, 128 mg; mineral oil, 3.75 mg.

4 Xylanase (Danisco, Dupont Nutrition & Bioscience) 40,000 U/g.

5 Phytase (Axtra PHY, Dupont Nutrition & Bioscience) 10,000 FTU.

6 All amino acids are expressed on standardized ileal digestible basis.

7 Glycine equivalents = glycine concentration + [serine concentration × 0.7143].

8 DEB: dietary electrolyte balance = K^+^ + Na^+^- Cl^−^.

#### Diet preparation

The test diets were formulated following near-infrared spectroscopy (NIR) of wheat and soybean meal via the AMINONir Advanced program (Evonik Operations GmbH, Hanau, Germany). Prior to mixing, wheat was ground using a hammer-mill with a 4.0 mm screen. The experimental diets were then steam-pelleted with a Palmer PP330 pellet press (Palmer Milling Engineering, Griffith, NSW, Australia) at a conditioning temperature of 80°C and a residence time of 14 seconds. After pelleting, the diets were cooled to ambient temperature in a vertical cooler. All diets contained feed phytase and xylanase. To determine digestibility coefficients of starch, protein (N), and amino acids, acid in soluble ash (Celite^TM^ World Minerals, Lompoc, CA) was incorporated into all diets at 20 g/kg as an inert marker.

### Data and sample collection, chemical analyses, calculations

#### Experiment 1

Growth performance (weight gain, feed intake, FCR) from 10 to 24 days post-hatch as grower and from 25 to 36 days as finisher was determined. Body weights and gender of dead or culled birds were recorded daily to correct feed intakes and adjust FCR calculations. Total excreta outputs and feed intakes were recorded from 28 to 30 days post-hatch to determine nutrient utilisation parameters by the ‘total excreta collection’ method as outlined by Truong et al. (2017).

On day 37 post-hatch, following overnight fasting, feed was offered to birds for 10 min, after which feed intake was measured and recorded. One drop of blood was collected from the brachial vein to determine glucose concentration by portable glucometer ACCU-CHEK produced by Roche Diabetes Care GmbH before eating, and then at 30 min interval until 240 min post-prandial. 3 ml blood samples (2 male birds per cage) was collected before offering diets as 0 min, and 180 min post-prandial from the brachial vein to determine the concentrations of insulin. Immediately following collection, blood samples were centrifuged, and decanted plasma samples were held at −80°C prior to analysis. Insulin concentration was determined following the instruction of Chicken Insulin ELISA Kit from CUSABIO technology LLC.

All birds were weighed and euthanised by intravenous injections of sodium pentobarbitone at day 38 post-hatch, the abdominal cavity opened, gender determined, and small intestines removed. Digesta samples were taken from the distal jejunum and distal ileum. The distal jejunum was defined by the mid-point between the end of the duodenal loop and Meckel’s diverticulum and the distal ileum was defined by the mid-point between Meckel’s diverticulum and the ileo-caecal junction. Digesta were gently expressed from these segments and samples from each pen were pooled, homogenized, freeze-dried, and ground through 0.5 mm screen to analyse starch, protein (N) and amino acids concentrations. Abdominal fat-pads were removed, weighed and relative abdominal fat-pad weights were calculated from final body weights. The *Pectoralis major, Pectoralis minor*, and leg quarters were dissected, and weights recorded against final body weights to determine relative weights or yields of carcass traits. The apparent jejunal and ileal digestibility coefficients of starch, protein (N) and amino acids were determined using the same methodology as described by Truong et al. (2017). The ratios of distal ileal S:P disappearance rate ratios were calculated as this eliminates the confounding influence of feed intake.

#### Experiment 2

On day 37 post-hatch, following overnight fasting, feed was offered to birds for 20 min, after which feed intake was measured and recorded. 3 mL systemic blood samples (2 birds, one male and one female) at 30, 120 and 200 min post-prandial were collected from brachial vein. Glucose levels were measured using Glucose HK kits from ThermoScientific. Different approaches were used to measure blood glucose in Experiment 1 and 2 due to differences in sampling time points. Experiment 1 involved more frequent sampling at multiple time points; therefore, glucose concentrations were measured immediately using a handheld glucometer. In contrast, Experiment 2 included fewer sampling points, allowing blood samples to be collected into tubes and analysed in the laboratory alongside other metabolites, including ammonia, fatty acids, and insulin. Although different methodologies were applied, previous evidence indicates that the results obtained from these approaches are comparable (Sopade and Gidley, 2009). Insulin concentration was analysed following Exp. 1. Twenty free amino acids were analysed by Waters Acquity UPLC method. Non-esterified fatty acids were analysed using Manual/RX Monza FA 115 kits following the instruction. Total area under the curve (tAUC) and incremental area under the curve (iAUC) were calculated according to Wolever (2004).

#### Statistical analysis

Experimental data in two studies were analysed by multiple-way analyses of covariance (ANCOVA) using the JMP Pro 16.0 software package (SAS Institute Inc. JMP Software, Cary, NC). The ratio of male bird numbers to total bird numbers in each cage was used as the covariant. The assumption of homogeneity of regression slopes was examined and confirmed by testing the association between treatment and the covariate. Linear and quadratic relationships and multiple linear regressions were established when deemed appropriate. The experimental unit was the cage means and a probability level of less than 5 % by Tukey test was considered statistically significant. Postprandial glucose, insulin, and metabolite concentrations were measured at multiple time points following feeding. Individual time points were analysed separately using the same statistical model described above. To provide an integrated assessment of the overall postprandial response, the AUC was calculated for each variable and analysed independently. Time was not included as a repeated factor in the statistical model, and treatment × time interactions were therefore not evaluated. Meal size and individual nutrient intakes were evaluated as potential covariates and included in the final models where significant correlations were observed between these variables and plasma metabolite concentrations. Intake data were also tabulated to demonstrate that their potential influence on the metabolic responses had been assessed. Direct comparison between the two studies should be interpreted with caution, as the duration of feeding experimental diets, bird age, and the sex of birds used for blood collection differed between the two studies.

## Results

The effect of dietary treatments on growth performance, carcass traits, and nutrient digestibility in Exp. 1 is shown in Table 3. During the grower phase, broilers offered the LCP diet had a 6.03 % lower weight gain (934 versus 994 g/bird) and a 6.54 % higher FCR (1.401 versus 1.315) compared with those offered the SCP diet, while no significant difference in FI was observed between the two treatments. There were no differences in BWG, FI, or FCR during the finisher or overall phase. Broilers offered the LCP diet exhibited lower breast yield compared with those offered the SCP diet. No differences in apparent starch and protein digestibilities were observed between the two treatments.

**Table 3:** The effect of dietary treatments on growth performance, carcass traits (g/kg) and nutrient digestibilities (Exp 1)*.

Treatment	10-24 days			25-36 days			10-36 days		
	BWG g/bird	FI g/bird	FCR	BWG g/bird	FI g/bird	FCR	BWG g/bird	FI g/bird	FCR
SCP	994^a^	1307	1.315^b^	1274	1944	1.531	2268	3249	1.435
LCP	934^b^	1308	1.401^a^	1280	1934	1.512	2214	3242	1.464
*SEM*	*13.3*	*16.1*	*0.0085*	*30.9*	*32.7*	*0.0237*	*38.9*	*43.8*	*0.0132*
P value	0.007	0.939	<0.001	0.894	0.844	0.578	0.353	0.913	0.146

						Apparent starch digestibilities		Apparent protein digestibilities	
	Fat pad	*P. Major*	*P. Minor*	Total breast	Leg	DJ	DI	DJ	DI
SCP	8.87	204^a^	40.9^a^	244^a^	190	0.889	0.912	0.744	0.821
LCP	9.73	190^b^	36.1^b^	226^b^	195	0.867	0.905	0.790	0.839
*SEM*	*0.527*	*3.1*	*0.74*	*3.6*	*2.9*	*0.0229*	*0.0251*	*0.0196*	*0.0096*
P value	0.279	0.009	<0.001	0.003	0.255	0.529	0.860	0.130	0.214

Cage means as the experimental unit, 10 replicate cages per treatment and 6 birds per replicate cage; DJ: distal jejunum; DI: distal ileum.

The effect of dietary treatments on nutrient disappearance rates at 38 days, nutrient utilization from 28 to 30 days, 37-day body weight, 10-minute intake of feed, starch, and protein in Exp. 1 are shown in Table 4. Broilers fed the LCP diet had significantly higher starch and NBAA intake than those fed the SCP diet. Broilers offered the LCP diet had a significant higher disappearance rate of starch in both the distal jejunum (DJ) and distal ileum (DI) compared with those offered the SCP diet. In contrast, disappearance rate of CP was significantly lower in the LCP treatment than in the SCP treatment in both DJ and DI. Accordingly, the disappearance rate ratio of starch to CP was greater in the LCP treatment than in the SCP treatment in both DJ and DI (P < 0.001). Boilers offered the LCP diet also had higher AME:GE and N retention than those offered the SCP diet.

**Table 4:** The dietary impact on nutrient disappearance rates at 38 days, nutrient utilisation from 28 to 30 days and 37-day body weight, intake of feed, starch, and protein within 10 min (Exp 1).

Treatment		Body weight	Feed intake	Starch intake	Protein intake	NBAA intake	Disappearance rate of starch g/b/d	
		g/bird	g/bird	g/bird 1	g/bird	g/bird	DJ	DI
SCP		2575	42.2	15.48^b^	8.96	0.231^c^	60.6^b^	62.2^b^
LCP		2520	45.7	20.31^a^	8.17	0.958^a^	69.6^a^	72.6^a^
*SEM*		*39.1*	*3.59*	*1.498*	*0.687*	*0.0619*	*1.79*	*1.88*
P value		0.357	0.512	0.042	0.445	<0.001	0.004	0.002

	Disappearance rate of CP g/b/d		Disappearance rate ratio of starch to CP		AME	AME:GE	N retention	AMEn
	DJ	DI	DJ	DI	MJ/kg		%	MJ/kg
SCP	29.9^a^	32.9^a^	2.03^b^	1.89^b^	12.73	0.773^b^	63.1^b^	11.03
LCP	25.7^b^	27.4^b^	2.72^a^	2.65^a^	12.54	0.792^a^	68.3^a^	11.01
*SEM*	*0.95*	*0.45*	*0.053*	*0.057*	*0.069*	*0.0043*	*0.81*	*0.071*
P value	0.007	<0.001	<0.001	<0.001	0.085	0.007	<0.001	0.828

1 Nutrientintake(g/bird)=feedintakeduiring10mins(g/bird)×analysednutrientcontent(g/kg)×1000; Cage means as the experimental unit, 10 replicate cages per treatment and 6 birds per replicate cage; NBAA: non-bound amino acids; DJ: distal jejunum; DI: distal ileum; CP: crude protein; AME: apparent metabolizable energy; GE: gross energy; AMEn: nitrogen-corrected AME.

The effects of dietary treatments on amino acid digestibility in the DJ and DI at 38 days post-hatch, as well as plasma glucose and insulin concentrations in systemic blood at 37 days post-hatch in Exp. 1 are presented in Table 5. In the DJ, digestibility of Arg, Ile, Lys, Met, Thr, Val, Glu, Gly, and Pro was significantly higher in broilers offered the LCP diet than in those offered the SCP diet (P < 0.05). In the DI, digestibility of Lys, Met, Thr, Val, Glu, and Gly were higher in the LCP treatment than that in the SCP treatment (P < 0.05). Insulin concentrations were significantly lower in the SCP treatment than in the LCP treatment at 180 min post-prandially. There were no differences in glucose concentrations at any time point or in the overall AUC.

**Table 5:** The effect of dietary treatments on digestibility of amino acids in the distal jejunum and ileum at day 38, post-prandial glucose and insulin concentration in systemic blood at 37 days post-hatch (Exp 1).

	Arg	His	Ile	Leu	Lys	Met		Phe	Thr	Val	Ala	Asp	Glu	Gly	Pro	Ser	Cys	Total
*Jejunum*																		
SCP	0.819^b^	0.775	0.761^b^	0.771	0.814^b^	0.892^a^		0.797	0.742^a^	0.741^a^	0.726	0.712	0.829^b^	0.718^b^	0.807^b^	0.754	0.652	0.781
LCP	0.873^a^	0.799	0.827^a^	0.824	0.870^a^	0.927^a^		0.835	0.813^a^	0.813^a^	0.737	0.719	0.876^a^	0.800^a^	0.857^a^	0.790	0.700	0.832
*SEM*	*0.0134*	*0.0185*	*0.0176*	*0.0177*	*0.0140*	*0.0085*		*0.0168*	*0.0179*	*0.0188*	*0.0233*	*0.0238*	*0.0139*	*0.0196*	*0.0152*	*0.0192*	*0.0259*	*0.0167*
P value	0.014	0.402	0.019	0.055	0.015	0.012		0.140	0.014	0.019	0.752	0.854	0.033	0.011	0.037	0.229	0.254	0.055
*Ileum*																		
SCP	0.894	0.851	0.843	0.851	0.866^b^	0.920^a^		0.885	0.810^b^	0.823^b^	0.795	0.805	0.893	0.804^b^	0.877^b^	0.835	0.769	0.855
LCP	0.912	0.851	0.872	0.871	0.894^a^	0.940^a^		0.897	0.848^a^	0.857^a^	0.781	0.782	0.914	0.849^a^	0.901^a^	0.838	0.792	0.875
*SEM*	*0.0062*	*0.0099*	*0.0098*	*0.0097*	*0.0081*	*0.0054*		*0.0090*	*0.0096*	*0.0104*	*0.0150*	*0.0128*	*0.0071*	*0.0094*	*0.0071*	*0.0104*	*0.0115*	*0.0088*
P value	0.057	0.988	0.061	0.185	0.028	0.022		0.374	0.019	0.038	0.524	0.229	0.057	0.004	0.032	0.862	0.185	0.141
*Plasma*	Insulin μIU/ml						Glucose mmol/L											
	0 min	180min	tAUC^1^	iAUC^2^		0 min		30 min	60 min	90 min	120min	150min	180min	210min	240min		tAUC	iAUC
SCP	7.26	6.32^b^	1245	−62		10.36		11.92	11.88	12.01	12.17	12.07	11.98	11.68	11.93		2845	358
LCP	7.07	7.67^a^	1143	−130		10.77		12.05	11.57	11.93	12.63	12.66	12.60	12.17	12.44		2916	332
*SEM*	*0.355*	*0.355*	*55.9*	*96.25*		*0.228*		*0.225*	*0.256*	*0.235*	*0.267*	*0.275*	*0.310*	*0.300*	*0.239*		*43.7*	*69.4*
P value	0.713	0.015	0.213	0.622		0.225		0.677	0.410	0.824	0.244	0.144	0.175	0.264	0.153		0.265	0.793

1 tAUC = Total area under the curve, iAUC = Incremental area under the curve, Cage means as the experimental unit, 10 replicate cages per treatment and 2 birds per replicate cage.

The interactive effects of dietary grain base and protein concentration on body weight at 38 days post-hatch and 20-minute intake of feed, starch, and protein in mixed-sex broiler chickens are presented in Table 6. There was an interactive effect between grain base and protein concentration for body weight (P = 0.031). In wheat-based diets, broilers offered the LCP diet had significantly lower body weight than those offered the SCP diet, whereas no difference was observed between the two dietary protein concentrations in sorghum-based diets. As a main factor, dietary CP reduction resulted in lower 20-minute intake of feed, starch and protein, regardless of the grain base.

**Table 6:** The impact of dietary grain base and protein concentration on body weight and 20-minute feed, starch and protein intake (g/bird) in straight-run broiler chickens (Exp 2).

Grain	Dietary CP	BW (g/bird)	FI (g/bird)	Starch intake (g/bird)^1^	Protein intake (g/bird)^2^
Wheat	SCP	2704^a^	84.6	32.5	16.7
	LCP	2509^b^	50.9	24.2	8.5
Sorghum	SCP	2726^a^	69.1	28.5	14.2
	LCP	2642^a^	30.9	14.5	5.6
*SEM*		*24.0*	*9.62*	*4.30*	*1.76*
Grain					
Wheat		2607	67.7	28.5	12.6
Sorghum		2684	50.0	21.5	9.9
CP					
SCP		2715	76.8^a^	30.5^a^	15.5^a^
LCP		2575	40.9^b^	19.5^b^	7.0^b^
P-value					
Grain		0.004	0.081	0.123	0.132
CP		<0.001	0.001	0.018	<0.001
Interaction		0.031	0.817	0.494	0.921

1 Starchintake(g/bird)=feedintakeduiring20mins(g/bird)×analysedstarchcontent(g/kg).

2 Proteinintake(g/bird)=feedintakeduiring20mins(g/bird)×analysedproteincontent(g/kg) Cage means as the experimental unit, 6 replicate cages per treatment and 6 birds per replicate cage.

The impact of dietary crude protein and grain type on post-prandial glucose, insulin, fatty acid and ammonia responses in Exp. 2 are shown in Table 7, Table 8. There was no significant interaction between grain type and dietary CP levels on glucose, fatty acid and insulin concentrations at the three time points. The only effect observed was that sorghum-based diets had higher glucose concentrations at 30 min, regardless of dietary CP levels. Wheat-based diets had lower ammonia levels at 120 min, and LCP diets, in general, had lower ammonia concentrations at both 120 and 200 min.

**Table 7:** The impact of dietary crude protein and grain type on post-prandial glucose and insulin response (Exp 2).

		Glucose (mmol/L)				Insulin (μIU/ml)			
Grain	CP	30 min	120 min	200 min	tAUC	30 min	120 min	200 min	tAUC
Wheat	SCP	13.3	12.9	12.6	2200	8.65	10.7	9.06	1573
	LCP	13.5	13.1	13.2	2243	8.40	10.8	8.94	1515
Sorghum	SCP	13.6	12.5	12.5	2185	7.83	11.7	9.25	1674
	LCP	14.3	13.1	12.9	2266	7.85	10.4	9.69	1625
*SEM*		0.27	0.36	0.28	41.3	0.426	0.84	0.436	51.6
Grain									
Wheat		13.4^b^	13.0	12.9	2222	8.53	10.7	8.99	1538
Sorghum		14.0^a^	12.8	12.7	2225	7.84	11.0	9.47	1650
CP									
SCP		13.5	12.7	12.6	2193	8.24	11.2	9.17	1634
LCP		13.9	13.1	13.0	2255	8.12	10.6	9.31	1570
P-value									
Grain		0.033	0.593	0.460	0.939	0.122	0.723	0.485	0.189
CP		0.143	0.284	0.158	0.151	0.785	0.535	0.777	0.513
Interaction		0.297	0.535	0.687	0.653	0.757	0.413	0.684	0.957

Cage means as the experimental unit, 10 replicate cages per treatment and 2 birds per replicate cage.

**Table 8:** The impact of dietary crude protein and grain type on post-prandial fatty acid and ammonia response (Exp 2).

		Fatty acid (mmol/L)				Ammonia (µg/L)			
Grain	CP	30 min	120 min	200 min	tAUC	30 min	120 min	200 min	tAUC
Wheat	SCP	0.528	0.386	0.443	70.3	5.31	4.79	4.92	803
	LCP	0.346	0.364	0.400	62.4	4.84	4.06	3.96	721
Sorghum	SCP	0.365	0.430	0.343	60.5	5.38	7.26	4.87	911
	LCP	0.320	0.342	0.388	59.0	4.82	4.55	4.09	703
*SEM*		*0.0684*	*0.0642*	*0.0524*	*7.59*	*0.432*	*0.545*	*0.383*	*90.8*
Grain									
Wheat		0.437	0.375	0.421	66.4	5.07	4.42^b^	4.44	762
Sorghum		0.343	0.386	0.365	59.8	5.10	5.90^a^	4.48	807
CP									
SCP		0.447	0.408	0.393	65.4	5.34	6.03^a^	4.89^a^	857
LCP		0.333	0.353	0.394	60.7	4.83	4.30^b^	4.03^b^	712
P-value									
Grain		0.195	0.867	0.300	0.396	0.946	0.019	0.922	0.628
CP		0.122	0.417	0.981	0.544	0.233	0.007	0.035	0.126
Interaction		0.337	0.627	0.405	0.679	0.919	0.100	0.804	0.497

Cage means as the experimental unit, 10 replicate cages per treatment and 2 birds per replicate cage.

The impact of dietary crude protein, grain type on amino acid plasma concentrations (µg/mL) at 30 min post-prandial are shown in Table 9. Dietary interactions were observed for His, Ser, Gly, Lys, Tyr, Met, Val, Ile, Leu, and Phe. The general trend was that reducing dietary CP in wheat-based diets increased plasma concentrations of these amino acids at 30 min, whereas in sorghum-based diets, concentrations either remained unchanged or decreased. Overall, reducing CP increased plasma concentrations of Gln and Pro, whereas wheat-based diets had higher levels of Pro and Trp. At 120 min (Table 10), most interactions disappeared. As main effects, wheat-based diets resulted in higher Pro, Cys, Val, and Ile concentrations, whereas LCP diets had lower concentrations of His, Cys, Tyr, branched-chain amino acids (BCAA), Phe, and Trp, but higher concentrations of Gln and Ala. Similarly, at 200 min (Table 11), LCP diets had lower concentrations of Gln, Arg, Cys, Lys, Tyr, Val, Ile, Leu and Phe compared with SCP diets. Wheat-based diets showed higher concentrations of His, Ser, Cys, Val, Ile and Leu, but lower concentrations of Ala. Overall, for the tAUC of each amino acid (Table 12), the lowest values for Ser, Tyr, Met, Leu, and Phe were observed in the sorghum-based LCP diet. As a main effect, LCP diets had lower His, Ala, and Cys concentrations but higher Gln tAUC. Wheat-based diets had higher Pro, Cys, Val, and Ile tAUC, but lower Ala tAUC.

**Table 9:** The impact of dietary crude protein, grain type on amino acid plasma concentrations (µg/mL) at 30 min post-prandial (Exp 2).

Grain	CP	His	Asn	Ser	Gln	Arg	Gly	Asp	Glu	Thr	Ala	Pro	Cys	Lys	Tyr	Met	Val	Ile	Leu	Phe	Trp	Total
Wheat	SCP	7.35^ab^	9.20	52.0^b^	188	82.0	43.9^c^	21.1	27.1	75.9	64.1	49.5	15.4	57.6	35.4^b^	16.6^b^	27.0^b^	14.7^b^	23.0^b^	19.8^a^	5.92	836^b^
	LCP	9.27^a^	6.26	65.9^a^	254	87.2	67.3^a^	23.8	27.9	108	63.9	74.8	14.9	63.7	32.6^b^	23.2^a^	36.4^a^	20.6^a^	34.3^a^	22.7^a^	6.67	1045^a^
Sorghum	SCP	8.64^a^	13.6	62.8^ab^	195	91.5	50.5^bc^	29.9	32.5	84.1	85.3	47.0	14.3	77.1	46.1^a^	21.4^ab^	27.7^b^	15.1^b^	30.2^a^	22.3^a^	5.68	960^ab^
	LCP	5.88^b^	9.76	51.1^b^	252	76.0	55.7^b^	22.0	23.6	85.5	69.2	55.1	11.7	65.0	23.6^c^	16.5^b^	23.7^b^	13.6^b^	22.0^b^	15.7^b^	5.21	906^ab^
*SEM*		*0.873*	*4.460*	*0.096*	*13.77*	*10.37*	*3.016*	*3.012*	*2.603*	*9.573*	*5.518*	*4.809*	*1.015*	*7.447*	*2.567*	*1.685*	*2.016*	*1.344*	*2.174*	*1.278*	*0.378*	*51.93*
Grain																						
Wheat		8.31	7.73	59.0	221	84.6	55.6	22.5	27.5	92.0	64.0^b^	62.1^a^	15.1	60.7	34.0	19.9	31.7	17.7	28.6	21.3	6.30^a^	940
Sorghum		7.26	11.7	56.9	224	83.7	53.1	25.9	28.1	84.8	77.2^a^	51.1^b^	13.0	71.1	34.8	19.0	25.7	14.3	26.1	19.0	5.44^b^	933
CP																						
SCP		7.99	11.41	57.4	192^b^	86.7	47.2	25.5	29.8	80.0	74.7	48.2^b^	14.8	67.3	40.7	19.0	27.4	14.9	26.6	21.1	5.80	898
LCP		7.57	8.01	58.5	253^a^	81.6	61.5	22.9	25.7	96.8	66.5	64.9^a^	13.3	64.4	28.1	19.9	30.1	17.1	28.2	19.2	5.94	975
*P-value*																						
Grain		0.246	0.394	0.612	0.846	0.935	0.424	0.263	0.823	0.462	0.028	0.034	0.052	0.181	0.747	0.596	0.009	0.024	0.257	0.091	0.037	0.887
CP		0.640	0.463	0.783	<0.001	0.626	<0.001	0.394	0.139	0.098	0.157	0.003	0.154	0.695	<0.001	0.623	0.199	0.125	0.468	0.161	0.718	0.154
Interaction		0.015	0.920	0.005	0.759	0.334	0.008	0.095	0.080	0.127	0.170	0.092	0.319	0.242	0.001	0.003	0.004	0.013	0.000	0.002	0.129	0.021

Cage means as the experimental unit, 10 replicate cages per treatment and 2 birds per replicate cage.

**Table 10:** The impact of dietary crude protein, grain type on amino acid plasma concentrations (µg/mL) at 120 min post-prandial (Exp 2).

Grain	CP	His	Asn	Ser	Gln	Arg	Gly	Asp	Glu	Thr	Ala	Pro	Cys	Lys	Tyr	Met	Val	Ile	Leu	Phe	Trp	Total
Wheat	SCP	6.95	18.17^a^	47.3	169	86.3	55.5	22.4	25.1	74.8	70.3	59.8	15.2	48.8	32.0	15.6	25.9	13.2	19.8	18.8	5.16	826
	LCP	5.67	2.66^c^	56.6	203	76.2	56.2	18.6	23.2	88.1	52.5	58.1	13.3	39.2	22.6	17.8	22.9	10.5	18.6	15.9	4.01	800
Sorghum	SCP	5.94	10.5^ab^	56.3	178	77.4	50.1	25.6	27.9	71.2	77.1	42.9	12.6	51.8	35.2	18.0	21.4	10.7	23.0	18.9	4.36	823
	LCP	4.28	10.73^b^	45.8	213	71.5	53.1	19.0	25.6	74.7	69.7	44.9	9.7	49.6	21.4	13.3	17.9	8.72	17.4	13.0	4.07	791
*SEM*		*0.629*	*2.489*	*3.825*	*11.83*	*8.289*	*4.876*	*3.523*	*2.313*	*8.301*	*5.648*	*5.648*	*1.015*	*4.064*	*1.784*	*1.593*	*1.585*	*0.829*	*1.416*	*0.912*	*0.342*	*46.92*
Grain																						
Wheat		6.31	10.42	51.9	186	81.2	55.8	20.5	24.2	81.5	61.4^b^	59.0^a^	14.2^a^	44.0	27.3	16.7	24.4^a^	11.8^a^	19.2	17.3	4.59	813
Sorghum		5.11	10.6	51.0	195	74.4	51.6	22.3	26.8	73.0	73.4^a^	43.9^b^	11.1^b^	50.7	28.3	15.7	19.6^b^	9.7^b^	20.2	16.0	4.21	807
CP																						
SCP		6.44^a^	14.35	51.8	173^b^	81.8	52.8	24.0	26.5	73.0	73.7^a^	51.4	13.9^a^	50.3	33.6^a^	16.8	23.7^a^	12.0^a^	21.4^a^	18.8^a^	4.76a	824
LCP		4.97^b^	6.69	51.2	208^a^	73.8	54.7	18.8	24.4	81.4	61.1^b^	51.5	11.5^b^	44.4	22.0^b^	15.5	20.4^b^	9.59^b^	18.0^b^	14.4^b^	4.04^b^	796
*P-value*																						
Grain		0.070	0.936	0.812	0.431	0.361	0.394	0.602	0.272	0.317	0.046	0.015	0.006	0.115	0.574	0.526	0.007	0.019	0.494	0.154	0.286	0.895
CP		0.026	0.005	0.870	0.007	0.270	0.697	0.146	0.360	0.309	0.033	0.979	0.026	0.149	<.001	0.421	0.046	0.008	0.021	<.001	0.043	0.537
Interaction		0.788	0.010	0.025	0.968	0.793	0.830	0.715	0.930	0.589	0.400	0.754	0.647	0.409	0.257	0.058	0.911	0.694	0.166	0.143	0.252	0.954

Cage means as the experimental unit, 10 replicate cages per treatment and 2 birds per replicate cage.

**Table 11:** The impact of dietary crude protein, grain type on amino acid plasma concentrations (µg/mL) at 200 min post-prandial (Exp 2).

Grain	CP	His	Asn	Ser	Gln	Arg	Gly	Asp	Glu	Thr	Ala	Pro	Cys	Lys	Tyr	Met	Val	Ile	Leu	Phe	Trp	Total
Wheat	SCP	5.71	10.06	54.2	161	72.0	46.3	17.2	25.7	69.8	59.1	45.0	15.9	43.7	27.1	14.1^a^	22.4	11.6	17.5	17.4	4.82	744
	LCP	4.33	5.09	55.0	182	57.6	47.9	14.7	24.6	77.0	47.7	42.1	12.7	32.1	20.4	14.5^a^	19.0	8.67	16.2	15.0	4.01	703
Sorghum	SCP	5.12	13.0	53.0	162	69.2	43.7	19.6	23.5	69.2	68.2	38.1	12.2	42.4	34.8	15.6^a^	19.0	9.38	22.8	17.8	3.86	743
	LCP	3.33	10.11	41.8	190	55.1	41.8	15.2	23.0	62.1	62.8	35.9	8.9	37.8	19.2	10.7^b^	14.6	7.04	17.9	12.8	4.19	676
*SEM*		*0.339*	*2.956*	*3.053*	*12.002*	*5.575*	*3.266*	*2.149*	*1.462*	*7.850*	*5.227*	*3.550*	*0.806*	*3.964*	*2.109*	*1.114*	*1.310*	*0.763*	*1.357*	*0.797*	*0.356*	*38.796*
Grain																						
Wheat		5.02^a^	7.57	54.6^a^	171	64.8	47.1	15.9	25.2	73.4	53.4^b^	43.6	14.3^a^	37.9	23.7	14.3	20.7^a^	10.1^a^	16.8^b^	16.2	4.41	724
Sorghum		4.22^b^	11.6	47.4^b^	176	62.2	42.7	17.4	23.2	65.6	65.5^a^	37.0	10.6^b^	40.1	27.0	13.1	16.8^b^	8.21^b^	20.4^a^	15.3	4.02	710
CP																						
SCP		5.41	11.53	53.6	162^b^	70.6^a^	45.0	18.4	24.6	69.5	63.6	41.6	14.1^a^	43.0^a^	31.0^a^	14.8	20.7^a^	10.5^a^	20.1^a^	17.6^a^	4.34	744
LCP		3.83	7.60	48.4	186^a^	56.4^b^	44.8	14.9	23.8	69.5	55.3	39.0	10.8^b^	35.0^b^	19.8^b^	12.6	16.8^b^	7.86^b^	17.0^b^	13.9^b^	4.10	690
P-value																						
Grain		0.030	0.192	0.029	0.691	0.636	0.192	0.496	0.201	0.329	0.031	0.079	0.000	0.587	0.136	0.315	0.008	0.020	0.017	0.270	0.286	0.723
CP		0.000	0.187	0.095	0.049	0.016	0.958	0.115	0.587	0.992	0.115	0.465	0.001	0.049	<.0001	0.049	0.006	0.002	0.030	0.000	0.493	0.166
Interaction		0.578	0.747	0.083	0.767	0.982	0.621	0.703	0.867	0.403	0.600	0.927	0.983	0.420	0.066	0.039	0.701	0.709	0.235	0.155	0.150	0.758

Cage means as the experimental unit, 10 replicate cages per treatment and 2 birds per replicate cage.

**Table 12:** The impact of dietary crude protein and grain type on the total area under the curve of postprandial amino acid response (mg/mL × min) (Exp 2).

Grain	CP	His	Asn	Ser	Glu	Arg	Gly	Asp	Gln	Thr	Ala	Pro	Cys	Lys	Tyr	Met	Val	Ile	Leu	Phe	Trp	Total
Wheat	SCP	1.14	1.80	8.55^ab^	4.34	13.9	8.54	3.60	28.8	12.9	11.0	9.02	2.62	8.46	5.24^b^	2.64^ab^	4.30	2.24	3.39^b^	3.17^a^	0.86	136.7
	LCP	1.06	0.62	9.97^a^	4.22	12.6	9.72	3.22	36.1	15.3	9.3	9.97	2.30	7.43	4.24^bc^	3.12^a^	4.32	2.15	3.76^ab^	2.96^a^	0.81	143.1
Sorghum	SCP	1.09	1.81	9.70^ab^	4.80	13.4	8.29	4.13	30.8	12.3	13.3	7.31	2.19	9.50	6.59^a^	3.10^a^	3.81	1.96	4.23^a^	3.32^a^	0.81	143.2
	LCP	0.79	1.75	7.88^b^	4.18	11.9	8.69	3.23	36.9	12.8	11.6	7.82	1.73	8.80	3.65^c^	2.32^b^	3.22	1.66	3.22^b^	2.34^b^	0.75	135.7
*SEM*		*0.082*	*0.443*	*0.499*	*0.245*	*1.03*	*0.507*	*0.464*	*1.85*	*1.29*	*0.81*	*0.680*	*0.137*	*0.680*	*0.272*	*0.209*	*0.200*	*0.105*	*0.168*	*0.110*	*0.045*	*5.96*
Grain																						
Wheat		1.10	1.21	9.26	4.28	13.2	9.13	3.41	32.4	14.1	10.1^b^	9.50^a^	2.46^a^	7.94	4.74	2.88	4.31^a^	2.20^a^	3.57	3.07	0.84	139.9
Sorghum		0.94	1.78	8.79	4.49	12.6	8.49	3.68	33.9	12.6	12.4^a^	7.56^b^	1.96^b^	9.15	5.12	2.71	3.51^b^	1.81^b^	3.72	2.83	0.78	139.5
CP																						
SCP		1.11^a^	1.81	9.13	4.57	13.6	8.41	3.87	29.8^b^	12.6	12.1^a^	8.16	2.40^a^	8.98	5.91	2.87	4.05	2.10	3.81	3.25	0.84	139.9
LCP		0.93^b^	1.18	8.92	4.20	12.3	9.21	3.22	36.5^a^	14.1	10.4^b^	8.89	2.02^b^	8.12	3.94	2.72	3.77	1.91	3.49	2.65	0.78	139.4
P-value																						
Grain		0.060	0.214	0.361	0.401	0.563	0.223	0.571	0.454	0.249	0.011	0.010	0.002	0.091	0.182	0.419	<.001	0.002	0.381	0.043	0.210	0.941
CP		0.033	0.177	0.691	0.143	0.207	0.132	0.181	0.002	0.261	0.047	0.296	0.010	0.219	<.001	0.476	0.173	0.084	0.076	<.001	0.214	0.933
Interaction		0.208	0.225	0.004	0.320	0.936	0.452	0.580	0.753	0.465	0.991	0.754	0.621	0.812	0.002	0.007	0.148	0.338	<.001	0.002	0.958	0.261

Cage means as the experimental unit, 10 replicate cages per treatment and 2 birds per replicate cage.

## Discussion

Predictably, reducing dietary CP with NBAA supplementation in Exp. 1 increased total tract N retention (63.1 % vs. 68.3 %). It also increased the apparent jejunal digestibility of all supplemented AA except Leu. These effects extended to the distal ileum for Lys, Met, Thr, Val, Gly, and Pro. However, there were no significant differences in the apparent protein digestibility of the distal jejunum and ileum between broilers offered the SCP and LCP diets at 38 days post-hatch. Consequently, dietary crude protein reduction did not affect final body weight, which contrasts with observations in the wheat‑based diet tested in Exp. 2. In Exp. 2, the wheat‑based reduced crude protein diet resulted in a 7.2 % reduction in final body weight (2704 vs. 2509 g/bird). Liu et al. (2021) suggested that the poor performance observed with reduced CP diets based on high‑protein grains may be attributable to increased grain, NBAA and reduced soybean meal inclusions compared to reduced CP diets based on low‑protein grains. The protein contents of the wheat in Exp. 1 and the sorghum in Exp. 2 were comparable (approximately 10–11 %), whereas the wheat used in Exp. 2 contained 12 % protein. Consequently, the same level of dietary CP reduction decreased soybean meal inclusion by approximately 75 % in the wheat-based diet in Exp. 2, but only 46 % in the wheat-based diet in Exp. 1 in compariosn to their cooresponding control SCP diets. The greater reduction in soybean meal and increased NBAA inclusion, which could cause an imbalance between protein-bound and non-bound amino acid supply, as well as between amino acid and glucose availabilities (Liu et al., 2021).

Another observation is that young birds were more vulnerable to dietary CP reduction, as observed in Exp. 1. Reducing CP from 21.5 % to 18.5 % in grower diets (10–24 days post‑hatch) significantly reduced body weight gain and compromised feed efficiency, although these differences did not persist into later stages. This suggests that early growth performance differences do not always carry through to later phases. For example, Xie et al. (2026) reported that significant body weight advantages associated with on‑farm hatching at 0 days (44 versus 55.6 g/bird), 14 days (546 versus 561 g/bird) and 28 days (1800 versus 1843 g/bird) post-hatch did not persist into the finisher phase (29–39 days). In contrast to the present study, Kandel et al. (2025b) reported that a two‑percentage‑point CP reduction, combined with the inclusion of legumes and by‑product protein meals to achieve 40 %, 59 %, 100 %, and 100 % replacement of soybean meal in the starter, grower, finisher, and withdrawal phases, respectively, did not compromise growth performance, particularly in younger birds during starter and grower phases. It was proposed that when the primary objective is to reduce reliance on imported soybean meal rather than to lower crude protein for nitrogen emission mitigation, a strategy combining NBAA with intact, alternative protein‑rich by‑product meals may be effective (Kandel et al., 2025b; Macelline et al., 2023). This approach may help avoid the limitation that excessive NBAA inclusion and CP reduction can lead to nitrogen deficiency or nutrient asynchrony (Macelline et al., 2022; Selle et al., 2020), while also preventing the need for high inclusion levels of alternative by‑product meals, which are often constrained by the presence of anti‑nutritional factors (Kandel et al., 2025a; Sadr et al., 2026).

Additionally, no correlations were observed between body weight and insulin concentrations in either of the two experiments. In Exp. 1, body weight was correlated to glucose concentrations at 120, 150, 180, 210, 240 mins. Insulin has received less attention in avian species than in mammals because birds are considered hyperglycemic and resistant to insulin (Braun and Sweazea, 2008). Ji et al. (2020) demonstrated differences in glucose response patterns between commercial broilers and Silky chickens following exogenous insulin injection, with Silky chickens exhibiting a greater capacity to regulate glucose homeostasis than broilers. However, the insulin dose used in that study was relatively high (2.3 IU/kg body weight, equivalent to 5750 µIU for a 2.5‑kg bird). This makes direct comparison with endogenous insulin concentrations measured in the present study (∼9 µIU/mL) difficult, particularly because total blood volume in chickens is not precisely defined. Nevertheless, the insulin challenge dose used by Ji et al. (2020) was undoubtedly substantially higher than physiological insulin concentrations typically occurring in modern broilers.

This highlights a broader challenge when comparing studies in the literature, as many investigations rely on glucose tolerance or insulin challenge models in which dietary formulation is not an influential factor, and supraphysiological doses of glucose or insulin are administered. An exception is the study by Adeleye et al. (2016), in which formulated poultry diets were used to examine the persist dietary effects on post‑prandial glucose responses. It was concluded that starch granule size and morphology may influence glucose responses in broiler chickens at 21 days post‑hatch; however, these effects were not associated with digesta kinetics or intestinal digesta transit rate. Apparent jejunal digestibility is often considered an indicator of digestive dynamics (Wang et al., 2026). In the present study, no treatment differences were observed in apparent jejunal starch digestibility in Exp. 1; however, birds fed the LCP diet showed higher insulin concentrations at 180 min post‑feeding. This pattern is consistent with the findings of Adeleye et al. (2016), in which glucose responses were detected independently of digesta kinetics. It should be noted that feed intake during the initial feeding window following fasting was not reported in Adeleye et al. (2016); hence, it is difficult to determine whether the observed glucose responses were driven by starch granule size per se or by differences in the quantity of starch intake. In the present Exp. 1, the 10‑min feed intake was positively correlated with insulin concentration at 180 min (r = 0.429, P = 0.018); there was no correlations between 10-min feed intake and blood glucose concentrations up to 90 min post‑feeding, whereas positive correlations were observed at 120 min (r = 0.404, P = 0.027), 150 min (r = 0.452, P = 0.012), and 210 min (r = 0.394, P = 0.031). Although birds are inherently hyperglycemic, Akiba et al. (1999) argued that insulin regulates blood glucose levels in a similar manner to mammals. In mammals, peripheral insulin resistance is characterized by hyperinsulinemia despite elevated or normal glucose concentration (Rudenski et al., 1991). In Exp. 2, despite differences in starch intake between SCP and LCP diets, regardless of dietary grain type, there were no differences in glucose and insulin responses. Furthermore, no associations were observed between starch intake and glucose or insulin responses. No direct correlations were detected between insulin and glucose concentrations at each time point in both Exp. 1 and Exp. 2. Collectively, The two experiments suggest that the relationship between insulin and glucose in chickens is not as straightforward as in mammals. However, the inconsistent relationships between meal size and postprandial glucose and insulin responses in Experiments 1 and 2 suggested that, to verify the direct impact of starch intake, a study specifically designed to administer titrated levels of starch and examine their effects should be considered. In contrast, meal size, reflected as feed intake during the defined offering period prior to blood collection, was positively correlated with blood ammonia (r = 0.494, P = 0.020), Phe (r = 0.445, P = 0.030), Cys (r = 0.567, P = 0.004), and Tyr (r = 0.594, P = 0.002) levels at 30 min in Exp 2. Unlike human clinical studies, where meal size is fixed, it is challenging to control intake in group-housed animals. In the present study, to ensure consistent timing, birds were offered feed for a defined period rather than a fixed amount. This potential confounding factor should be considered in future experimental designs.

One motivation for investigating glucose response patterns in broiler chickens offered LCP diets is to better understand the mechanisms underlying the increased abdominal fat deposition observed in birds fed LCP diets, even when growth performance is not compromised (Chrystal et al., 2021; Wang et al., 2025). In Exp. 2, no correlations were observed between blood glucose, insulin, fatty acid, and ammonia concentrations at any time point. There were also no correlations between fat pad weights and these variables. Additionally, dietary CP level had no effect on plasma fatty acid concentrations. In least‑cost feed formulation, LCP diets often contain higher starch and lower fat levels in order to maintain iso‑energetic diets. Dietary fat reduced post-prandial glucose in a dose-dependent response in human (Bell et al., 2019), and delayed the glucose peak by 29 mins (Bell et al., 2015). Large protein load resulted in delayed and maintained post-prandial glucose concentration in human (Paterson et al., 2016). This could not be verified in the present study, as differences in protein intake between LCP and SCP diets did not alter glucose responses.

In the present study, systemic blood was collected due to the ease of sampling. It is important to recognise that systemic amino acid (AA) concentrations are the result of intestinal absorption, enterocyte metabolism, and hepatic processing. Following the meal and digestion, amino acids are absorbed across the intestinal epithelium into enterocytes; however, not all absorbed amino acids are directly transferred into the portal circulation for systemic protein accretion and a substantial proportion of dietary amino acids undergoing intracellular metabolism before leaving the gut (Wu, 1998). Amino acids that enter the portal vein are subsequently transported to the liver, which plays a central regulatory role by directing amino acids toward protein synthesis, oxidation, or conversion into other metabolites before releasing the remaining amino acids into the systemic circulation, where they become available for uptake by peripheral tissues such as muscle (Wu et al., 2014). Indeed, Truong et al. (2017) reported a 12.7 % reduction when comparing amino acid concentrations between the portal and systemic circulations and the summit diet containing higher NBAA led to higher concentrations of Ile, Met, Thr, Pro and Tyr. These observations contrast the present study where LCP diets, regardless of grain type, reduced Arg, Cys, Lys, Tyr, Val, Ile, Leu, and Phe concentrations at 200 min. It is unclear whether these differences are due to the fact that, in the Truong et al. (2017) study, the analysed dietary CP and total nitrogen pool were not altered, but only the protein source was changed, whereas in the present study, the analysed CP content decreased from 202 g/kg in the SCP diets to 174 g/kg in the LCP diets. Interestingly, most dietary interactions affecting AA concentrations occurred at 30 min postprandially, where the wheat-based LCP diet showed the highest Gly, Val and Ile concentrations. However, these differences disappeared by 200 min. This may indicate greater tissue uptake of Gly, Val and Ile in the wheat-based LCP diet. Such a hypothesis is somewhat puzzling, as compromised growth performance and body weight were observed with this diet (Wang et al., 2025).

Branched-chain amino acids are largely not metabolised in the liver due to low hepatic branched-chain aminotransferase activity; consequently, they pass through the liver and are released into the systemic circulation, where they are preferentially taken up and utilised by skeletal muscle (Harper et al., 1984). This aligns with our observation that the wheat-based LCP diet had the highest Val and Ile concentrations at 30 min. However, this increase disappeared over time, merging into main effects whereby wheat-based diets, regardless of SCP or LCP, showed higher Val and Ile concentrations in blood at 120 and 200 min. The initially elevated BCAA concentrations at 30 min may be attributed to higher dietary BCAA supplementation in the wheat-based LCP diet (Table 2). However, the persistently higher BCAA concentrations at later time points may suggest that BCAA uptake by muscle was less efficient in wheat-based diets once metabolic conditions became more stable. It is unclear whether this is caused by limited amino acid transporter capacity in muscle or by an imbalance of other amino acids required for muscle protein synthesis.

Another noticeable dietary difference in Exp. 2 is that the sorghum-based LCP diet contained a much higher level of added glutamic acid to restore its concentration to that of the SCP diets. Sorghum naturally contains lower glutamic acid than wheat (Ravindran et al., 2005); consequently, the sorghum-based LCP diet included 8.8 g/kg of added glutamic acid, whereas the wheat-based diet contained only 1 g/kg. Glutamate may be extensively catabolised in avian enterocytes to provide energy for the gut (Selle et al., 2024). At 30 min, there was a significant increase in plasma glutamine concentrations (192 vs. 253 µg/mL), and this difference persisted at 120 and 200 min, which was also reflected in the total area under the curve (tAUC) for glutamine. One proposed hypothesis to explain the better growth performance observed in the sorghum-based LCP diet, compared with the wheat-based LCP diet, is that the elevated dietary glutamic acid level may have helped optimise energy supply and function of the small intestine. Overall, these findings suggest that focusing solely on amino acid responses in systemic blood may not provide a complete understanding of amino acid metabolism in LCP diets. In the present study, no direct correlation was observed between circulating amino acid concentrations and growth performance. Future research should therefore incorporate more localised and mechanistic assessments, such as intestinal and hepatic metabolism and tissue-specific amino acid uptake, to better understand the limitations of amino acid utilisation in LCP diets.

## Conclusions

Reducing dietary crude protein with NBAA supplementation improved nitrogen utilisation and apparent amino acid digestibility; however, these benefits did not consistently result in improved growth performance. Younger birds were more sensitive to dietary CP reduction, as reduced growth performance was observed during the grower phase, although these early effects did not necessarily persist to market age. There were no clear relationships between glucose, insulin, and fat pad weights, which differed from our hypothesis. However, the absence of more comprehensive lipid-related measurements, such as lipogenic enzyme activities, limited definitive interpretation. Therefore, it remains inconclusive whether alterations in glycaemic responses in chickens contribute to variations in fat deposition. The null hypothesis that grain type would not influence postprandial amino acid responses under LCP conditions was rejected, as a dietary interaction effect on amino acid concentrations was observed at 30 min. However, the present study is limited in its interpretation, as amino acid responses in systemic blood alone may not provide a complete understanding of amino acid metabolism in LCP diets. More localised and mechanistic assessments should be considered in future studies, with particular attention to the influence of meal size on outcomes.

## Disclosures

The authors declare that the research was conducted in the absence of any commercial or financial relationships that could be construed as a potential conflict of interest.

## Author Contribution

Mengzhu Wang led the study design, data collection and analysis, figure preparation, and drafting the original manuscript. Shemil P. Macelline assisted with study design, data collection and analysis, supervised the research, and contributed to manuscript preparation and revision. Mehdi Toghyani contributed to feed formulation and finalised the manuscript. Sonia Y. Liu conceived and supervised the project, secured research funding, contributed to the investigation, and assisted in writing and revising the manuscript.

## CRediT authorship contribution statement

**Mengzhu Wang:** Writing – review & editing, Writing – original draft, Visualization, Methodology, Investigation, Formal analysis, Data curation, Conceptualization. **Shemil P. Macelline:** Writing – review & editing, Writing – original draft, Supervision, Methodology, Investigation, Formal analysis, Data curation. **Mehdi Toghyani:** Writing – review & editing, Methodology. **Sonia Y. Liu:** Writing – review & editing, Writing – original draft, Supervision, Investigation, Funding acquisition, Conceptualization.
